# Crowding-Out Effect of Out-of-Pocket Health Expenditures on Consumption Among Households in Mongolia

**DOI:** 10.34172/ijhpm.2021.91

**Published:** 2021-08-25

**Authors:** Ochirbat Batbold, Tuvshin Banzragch, Davaatseren Davaajargal, Christy Pu

**Affiliations:** ^1^Ach Medical University, Ulaanbaatar, Mongolia.; ^2^Institute of Public Health, National Yang Ming Chiao Tung University, Taipei, Taiwan.; ^3^School of Medicine, Etugen University, Ulaanbaatar, Mongolia.; ^4^Mongolian Institute of Certified Public Accountants, Ulaanbaatar, Mongolia.; ^5^National Statistics Office of Mongolia, Ulaanbaatar, Mongolia.

**Keywords:** Out-of-Pocket, Crowding Out, Private Health Insurance, Social Health Insurance, Financial Protection, Mongolia

## Abstract

**Background:** High out-of-pocket (OOP) health expenditures are a common problem in developing countries. Studies rarely investigate the crowding-out effect of OOP health expenditures on other areas of household consumption. OOP health costs are a colossal burden on families and can lead to adjustments in other areas of consumption to cope with these costs.

**Methods:** This cross-sectional study used self-reported household consumption data from the nationally representative Household Socioeconomic Survey (HSES), collected in 2018 by the National Statistical Office of Mongolia. We estimated a quadratic conditional Engel curves system to determine intrahousehold resource allocation among 12 consumption variables. The 3-stage least squared method was used to deal with heteroscedasticity and endogeneity problems to estimate the causal crowding-out effect of OOP.

**Results:** The mean monthly OOP health expenditure per household was ₮64 673 (standard deviation [SD]=259 604), representing approximately 6.9% of total household expenditures. OOP health expenditures were associated with crowding out durables, communication, transportation, and rent, and with crowding in education and heating for all households. The crowding-out effect of ₮10 000 in OOP health expenditures was the largest for food (₮5149, 95% CI=−8582; −1695) and crowding-in effect was largest in heating (₮2691, 95% CI=737; 4649) in the lowest-income households. The effect of heating was more than 10 times greater than that in highest-income households (₮261, 95% CI=66; 454); in the highest-income households, food had a crowding-in effect (₮179, 95% CI=-445; 802) in absolute amounts. In terms of absolute amount, the crowding-out effect for food was up to 5 times greater in households without social health insurance (SHI) than in those with SHI.

**Conclusion:** Our findings suggest that Mongolia’s OOP health expenses are associated with reduced essential expenditure on items such as durables, communication, transportation, rent, and food. The effect varies by household income level and SHI status, and the lowest-income families were most vulnerable. SHI in Mongolia may not protect households from large OOP health expenditures.

## Background

**Key Messages**

** Implications for policy makers**

- Whether a country can protect its citizens from high out-of-pocket (OOP) costs has become an essential policy question for all governments.
- Despite their importance, OOP health expenditures have not received much attention in developing countries as a result of a lack of data.
- Mongolia’s OOP health expenses are associated with reduced essential expenditure on items such as durables, communication, transportation, rent, and food.
- Social health insurance (SHI) in Mongolia may not protect households from large OOP health expenditures.

** Implications for the public**

High out-of-pocket (OOP) often poses significant financial burdens for households. In Mongolia, the contribution of social health insurance (SHI) to total health expenditure remains low. Our study examines the adverse impacts of OOP health expenditures on households and provides an examination of the country’s universal health coverage (UHC) progress from a household crowding-out perspective. In countries where there is high OOP, the government should design policies to alleviate the financial burden for its citizens. Our study facilitates such policy development and hence has the potential to benefit Mongolian households as well as households from countries with similar healthcare system by reducing OOP burden.

Achieving universal health coverage (UHC) has become an essential health policy goal worldwide.^1^ The two core components of UHC are coverage of the population with high-quality and essential health services, and provide financial protection. The latter is the key to reducing OOP health expenditures for households.^2^ Out-of-pocket (OOP) healthcare expenditure is a prominent policy concern due to the financial burden it imposes. The importance of UHC is reflected in the United Nations Sustainable Development Goals 3 agenda and the Thirteenth General Programme of Work of the World Health Organization (WHO).^3,4^ The policy importance of OOP health expenditures globally is evidenced by the fact that the SDGs include an indicator for measuring OOP health expenditures relative to a family’s means (indicator 3.8.2).^5^

 The WHO estimated that in 2015, 926.6 million people incurred catastrophic OOP health expenditures, with OOP health expenses exceeding 10% of their household budget; OOP health expenditures exceeded 25% of the household budget of 208.7 million people.^6^ Asian and middle-income countries had the highest percentages of households facing catastrophic health expenditures.^6^ Studies assessing OOP health expenditures, their impact on households, and awareness and attitudes regarding health insurance in populations have produced differing results.^7,8^ However, OOP health expenditures pose a colossal burden for families and can lead to subsequent impoverishment.^9^ People of lower economic status, households with older people, and households located in rural areas^10^ are more likely to incur higher medical costs and fall deeper into poverty.^8,11^

 Mongolia is a landlocked country located between Russia to the north and China to the south. Studies on the healthcare system and OOP health expenditures in Mongolia are scant. Like other developing countries, Mongolia’s health financing reforms are guided by the UHC aim to reduce inequality and expand financial protection.^12-14^ Three main options are used in Mongolia to finance national healthcare expenditures, namely state budgets, insurance contributions, and direct OOP health payments^15^ by households, as in other low- and middle-income countries (LMICs).^16,17^

###  Mongolia’s Healthcare System

 Before 1990, Mongolia had a Semashko-style centralized healthcare system,^18^ where the government was wholly in charge of health service delivery and financing.^19^ It provided everybody access to universal, free of charge healthcare. However, after 1990, Mongolia enacted political and economic reforms to move toward a democratic system and a market-oriented economy.^18^ Health financing became a “problem” as it was in many former Soviet Union countries during their transition period.^20^ Rural healthcare is highly resource intensive, especially in countries with a low population density.^18^ In 1994, Mongolia’s government successfully introduced a new social health insurance (SHI)^21^ system to promote equitable access to healthcare and to provide financial protection, especially for low-income and vulnerable groups. Their SHI’s population coverage reached 97.7% in 2014 and 98.6% in 2016,^22,23^ with insurance made to be mandatory for all Mongolian citizens. Under the health insurance law, the SHI premium for employees is 4% of their salary, which is shared equally between employers and employees. The government fully subsidizes vulnerable groups and specific groups such as children (age <18 years), pensioners, mothers caring for new-born children, military personnel, and low-income populations. The premium rate is equal to 1% to 2% of the minimum wage for the remaining population.^23^ Mongolian SHI’s benefits package includes primary inpatient services (covering 85%-90% of expenses) and a limited number of outpatient services. It also covers part of the cost of essential medicines^14,23,24^ (40%-83% of drug expenses).^14^

 Despite the high SHI coverage levels, Mongolian households had a high OOP health expenditure share (32.36% of all health expenditures) in 2018.^25^ Mongolia has failed to prevent catastrophic health expenditures and medical impoverishment, unlike other LMICs with high insurance coverage.^11^ Researchers have determined that in 2016, 5.5% of Mongolian households (approximately 20 000) incurred catastrophic health expenditures, amounting to 10% of the total household income,^23^ reflecting a devastating level of health expenditure.^22,26,27^ OOP health expenditures have contributed to an 8% increase in poverty; the main drivers of this financial distress were expenditures on outpatient services, including diagnostics and drugs.^14^ Mongolia has failed to achieve the UHC goal of providing comprehensive healthcare services of acceptable quality that do not create financial hardship, even with the high SHI population coverage.^19^ Financial protections are inadequate, and increasing OOP healthcare expenses are a growing public and policy concern.

 Most studies have focused on the effect of OOP health costs on impoverishment without looking at their crowding-out effect. Households change the distribution of their consumption pattern to cope with OOP health expenditures. When the share for a particular good increases, it is deemed a crowding-in effect, and when it decreases, it is deemed a crowding-out effect. The crowding-out effect of OOP health expenses on a household’s other expenditures should not be overlooked because OOP health costs can lead to a reduction in spending on essential goods, which leads to the deterioration of living standards and exacerbates the impact of poverty.^28^ Expenditure patterns affect a household’s resource allocation, frequently with adverse consequences (eg, lower expenditure on essential items such as food and education),^29^ especially among vulnerable families with lower income levels.^30^ The crowding-out effect of OOP health expenses on these households may be particularly prominent.

 The purpose of the present study was to estimate the crowding-out effect of OOP health expenditures among Mongolian households with and without SHI and to evaluate whether the crowding-out effect differs among households of different income levels. More specifically, we investigated how the composition of household consumption for 12 expenditure categories changes as a result of OOP health expenditures and according to SHI status and income level in Mongolia. We compared households with and without SHI to determine whether SHI provides sufficient financial protection for citizens and compared the crowding-out effect for different income levels because income is the key determinant of financial vulnerability. We hypothesized that OOP health expenditures can crowd out essential items such as food, education, clothing, and transportation despite a high percentage of SHI coverage as a result of high existing OOP health expenses, a low-density population, and a low quality of health services in rural areas.

## Methods

 The data used in this study were obtained from the nationwide cross-sectional Household Socioeconomic Survey (HSES) conducted in 2018 by the National Statistical Office of Mongolia. The HSES is a nationally representative survey that estimates and monitors the country’s poverty level and living standards. It aims to update consumption weights for consumer price index baskets and to estimate private consumption expenditures for national accounts. Similar national household expenditure surveys have often been used to study household spending behavior.^29,30^ The data were collected over 12 months. The main comprehensive form of the survey for poverty estimation has been implemented biannually since 2012. For data collection, a computer-assisted personal interviewing approach has been used since 2014. In the 2018 HSES, 99.8% of the selected households (16 454 of 16 488) participated, with 1374 households surveyed each month.

 We used data from the 2018 survey because these data were the most recent available. Twelve household consumption categories were included: alcohol, tobacco, clothing, communication, durables, education, food, heating, rent, transportation, utilities, and other. We retained these categories in our analysis. Respondents were asked to report their consumption in these categories using a 30-day reference period.

 The respondents were also asked to report their outpatient visits and associated OOP health expenses with a 30-day reference period and, in a separate question, were requested to provide information of their drug expenditures. For inpatient care, the respondents were asked to report OOP health costs for the previous year; thus, we divided the costs by 12 to make them monthly, for consistency with previous studies.^14,30^ We thus derived the total healthcare OOP health expenses by summing up outpatient, inpatient, and pharmaceutical costs.

###  Statistical Methods

 Because the share of OOP health expenditure in a household budget is likely to be endogenous, we estimated the crowding-out effect using a 3-stage least squares strategy. We first estimated an Engel demand function, which assumed that OOP health costs were prefixed and that households made consumption decisions for other expenditure categories after predetermining OOP health costs. This is not an unreasonable assumption; previous empirical results suggest that this is often the case.^31^ The Engel curve we estimated is specified as follows^29,31^:


$$w_{ij}=a_{1i}+a_{2i}p_{nj}q_{nj}+\delta_{i}H_{j}+\beta_{1i}lnM_{j}+\beta 2_{i}{\left(lnM_{j}\right)}^{2}+\varepsilon_{ij}$$

where *w*_ij_ is the budget share by household *j* to the expenditure category *i*with budget *M*, and where *M* is calculated as total expenditures excluding OOP. $p\_ni\;\bar{q}\_nj$ is the OOP health expenditure for household *j*. *H* is a vector representing household characteristics, and *ε* is the random error term. This equation cannot be estimated using an ordinary least squares method because the variables *pq*and *lnM*(and *lnM*^2^)are likely endogenous due to the simultaneity involved. We thus instrumented these variables with*ln( total income)* and their squared values, SHI status and the share of young children and old adults. These are reasonable instrumental variables because it is fair to assume that households make consumption decisions for other expenditure categories after predetermining OOP and that, thus, each expenditure’s share (other than OOP) is also determined ex post. These instrumental variables are commonly used for studying crowding-out effects.^30,31^ SHI status and the share of young children and old adults are assumed to be correlated with OOP but uncorrelated directly with the share of expenditure categories.

 In our model, *H* includes the age of the household head, household size, sex of the household head, at least one household member being >65 years old, number of children, family structure (nuclear family, household of one resident living alone, a married couple with no other family members, single-parent family, or other), educational attainment of the household head (no formal education, primary, some secondary, complete secondary, vocational/special vocation, or college or higher), occupation of the household head (wage employed, herder, self-employed, unemployed, or other), marital status of the household head (married, single, common law, divorced/separated, or widowed), and region of residence (Ulaanbaatar, Highlands, Central, East, and West).

 Income was self-reported. We adjusted the income according to the number of adults and children using an equivalence scale proposed by Aronson et al^32^:


$$e_{h}={\left(A_{h}+\phi K_{h}\right)}^{\theta }$$

where *e*_h_ is the equivalence factor for household* h*,* A* is the number of adults, and *K* is the number of children in household *h. *We set the parameters ɸ equal to 0.5 and θ equal to 0.4684 as proposed by Koch.^33^ This adjustment was necessary to ensure that an accurate reflection of the household’s financial situation was obtained, as the number of people in a household can create economies of scale.

 A separate Engel curve was estimated for each expenditure category. The system of equations was estimated using seemingly unrelated regression with instrumental variables in accordance with the 3-stage least squares strategy.^29^ We stratified our analysis by tertile for household income and whether the household head was covered by SHI. The crowding-out effect, expressed in Mongolian tugrik (₮) terms, was calculated through multiplication of α_2i_ by total household expenditure for each stratified group (income and SHI status). We present the marginal effect per ₮10 000 increase in OOP health expenses on other household expenditure categories by household income level and SHI statuses.

## Results

Table 1 presents the household characteristics by household head SHI status. The majority (81.4%) of household heads were covered by SHI (hereafter, households with SHI). Households with SHI had, on average, higher OOP health costs compared with households without SHI (₮71 116 [SD = 257 244] and ₮36 438 [SD = 172 532], respectively) and had, on average, higher educational attainment.

**Table 1:** Profile of Household Survey Participants

**Variable/Categories**	**Household Head Have SHI**			***P*** ** Value**
	**Total (n = 16 454)**	**Yes (n = 13 397)**	**No (n = 3057)**	
	**No. (%)**	**No. (%)**	**No. (%)**	
Out-of-pocket health expenditure, mean (SD)	64 673 (259 604)	71 116 (275 244)	36 438 (172 532)	<.001
Age of household head, mean (SD) [range]	46.65 (14.34) [17-98]	48.22 (14.85) [17-98]	39.73 (8.98) [19-59]	<.001
Household size, mean (SD) [range]	3.50 (1.72) [1-16]	3.39 (1.70) [1-16]	3.99 (1.73) [1-13]	<.001
Head of household is male	12561 (76.34)	9836 (73.42)	2725 (89.14)	<.001
Household have at least one member >65-year-old	2150 (13.07)	2058 (15.36)	92 (3.01)	<.001
Number of children, mean (SD) [range]	1.35 (1.31) [0-9]	1.24 (1.28) [0-9]	1.79 (1.36) [0-7]	<.001
Family structure				<.001
Nuclear family	7769 (47.22)	5905 (44.08)	1864 (60.97)	
Household head living alone	2288 (13.91)	2008 (14.99)	280 (9.16)	
Married couple with no other family members	1665 (10.12)	1516 (11.32)	149 (4.87)	
Single parent family	1795 (10.91)	1534 (11.45)	261 (8.54)	
Other	2937 (17.85)	2434 (18.17)	503 (16.45)	
Education status of the household head				<.001
No formal education	768 (4.67)	565 (4.22)	203 (6.64)	
Primary	1663 (10.11)	1314 (9.81)	349 (11.42)	
Secondary	2837 (17.24)	2186 (16.32)	651 (21.30)	
Complete secondary	4142 (25.17)	3205 (23.92)	937 (30.65)	
Vocational/ special vocational	3685 (22.40)	3057 (22.82)	628 (20.54)	
College or above	3359 (20.41)	3070 (22.92)	289 (9.45)	
Occupation status of the household head				<.001
Wage employed	6881 (41.82)	5767 (43.05)	1114 (36.44)	
Herder	3023 (18.37)	2250 (16.79)	773 (25.29)	
Self employed	1439 (8.75)	938 (7.00)	501 (16.39)	
Unemployed	4991 (30.33)	4362 (32.56)	629 (20.58)	
Other	120 (0.73)	80 (0.60)	40 (1.31)	
Marital status of the household head				<.001
Married	10 073 (61.22)	8043 (60.04)	2030 (66.40)	
Single	1233 (7.49)	960 (7.17)	273 (8.93)	
Common law	1244 (7.56)	895 (6.68)	349 (11.42)	
Divorced/separated	1154 (7.01)	914 (6.82)	240 (7.85)	
Widowed	2750 (16.71)	2585 (19.30)	165 (5.40)	
Region				<.001
Ulaanbaatar	3573 (21.72)	2914 (21.75)	659 (21.56)	
Highlands	3911 (23.77)	3191 (23.82)	720 (23.55)	
Central	3980 (24.19)	3337 (24.91)	643 (21.03)	
East	1871 (11.37)	1504 (11.23)	367 (12.01)	
West	3119 (18.96)	2451 (18.30)	668 (21.85)	

Abbreviations: SD, standard deviation; SHI, social health insurance.

 The average consumption and the share of each expenditure category by income group are presented in Table 2. OOP health costs, on average, accounted for approximately 6.9% of total expenditures for all income groups. Households with higher incomes also had higher total consumption and higher OOP healthcare costs. The transportation variable among high-income households had the highest share at 10.5% of total expenditures, exceeding the share among the lowest- and middle-income households (4.8% and 7.6%, respectively). However, the lowest-income households spent more on food (₮206 049 [SD = 92 767]) at 39.1% and heating (₮36 451 [SD = 35 667]) at 6.9% of total expenditures compared with high- and middle-income households.

**Table 2:** Average Consumption and Category Shares According to Household Income Level

	**Total (n=16454)**		**Household Equalized Income**					
			**Lowest (n = 5485)**		**Middle (n = 5485)**		**Highest (n** **= 5484)**		***P*** ** Value**
	**Mean (SD)**	**%**	**Mean (SD)**	**%**	**Mean (SD)**	**%**	**Mean (SD)**	**%**	
Household income	1 121 179 (874 341)		470 674 (136 528)		922 719 (142 821)		1 970 297 (1 034 933)		<.001
Household income^a^	645 046 (485 184)		331 858 (114 539)		537 667 (145 043)		1 065 691 (620 956)		<.001
Consumption expenditures									
Outpatient services	35 133 (216 429)	3.7	19 343 (107 449)	3.7	30 129 (164 215)	3.5	55 932 (318 328)	3.9	<.001
Impatient services	12 065 (72 671)	1.3	4771 (22 512)	0.9	10 112 (49 407)	1.2	21 313 (112 944)	1.5	<.001
Drug expenditure	17 475 (69 177)	1.9	9192 (37 224)	1.7	17 178 (81 629)	2.0	26 057 (78 530)	1.8	<.001
Alcohol	3349 (16 467)	0.4	1799 (9805)	0.3	2684 (14 016)	0.3	5563 (22 656)	0.4	<.001
Smoking	9973 (22 594)	1.1	7033 (17 006)	1.3	10 060 (20 829)	1.2	12 827 (28 139)	0.9	<.001
Clothes	143 829 (158 262)	15.3	73 833 (80 647)	14.0	131 204 (117 664)	15.3	226 465 (207 163)	15.8	<.001
Communication	35 929 (42 571)	3.8	16 889 (18 113)	3.2	31 052 (25 511)	3.6	59 851 (59 164)	4.2	<.001
Durable	30 593 (50 230)	3.3	11 895 (26 567)	2.3	23 622 (27 334)	2.7	56 267 (71 134)	3.9	<.001
Education	53 227 (150 041)	5.7	17 025 (60 706)	3.2	45 945 (117 562)	5.3	96 718 (216 305)	6.8	<.001
Food	295 159 (155 803)	31.4	206 049 (92 767)	39.1	290 995 (118 966)	33.9	388 449 (182 794)	27.1	<.001
Heating	40 664 (39 629)	4.3	36 451 (35 667)	6.9	41 953 (38 200)	4.9	43 589 (44 187)	3.0	<.001
Other	99 044 (111 049)	10.5	50 361 (39 832)	9.6	86 677 (73 121)	10.3	153 453 (157 261)	11.1	<.001
Rent	55 054 (83 218)	5.9	27 779 (47 946)	5.3	45 530 (67 055)	5.3	91 860 (108 603)	6.4	<.001
Transport	80 279 (160 817)	8.5	25 476 (54 284)	4.8	64 883 (92 316)	7.6	150 491 (240 752)	10.5	<.001
Utility	27 254 (27 702)	2.9	18 634 (17 018)	3.5	25 598 (22 078)	3.0	37 530 (36 645)	2.6	<.001
Total	939 026 (658 863)	100.0	526 529 (287 012)	100.0	859 025 (394 666)	100.0	1 431 613 (803 202)	100.0	<.001

Abbreviation: SD, standard deviation. ^a^ Equalized income.

 This was true for all 3 types of OOP health expenses. OOP health expenditures on outpatient visits were significantly higher than OOP inpatient and drug expenditures. This was not unexpected; as mentioned previously, SHI covers inpatient but not outpatient care. In our sample, 89.0% of high-income households had a household head covered by SHI, whereas the corresponding proportions for low- and middle-income households were 72.1% and 83.2%, respectively. For all income groups, food share of total expenditure was the largest category, followed by clothing.

 The crowding-out effect of OOP health costs on other categories of expenditure by SHI is presented in Table 3. On average, every ₮10 000 crowded out ₮1493 of expenditure on durables and ₮1765 of expenditure on transportation. A negative crowding-out effect was also observed for the communication (₮765), food (₮433), rent (₮391), and other (₮1052) categories. When we stratified the analysis by SHI status, the crowding-out effect for clothing was observed only for households with SHI. Larger crowding-out effects were observed among households without SHI for durables, food, and transportation compared with households with SHI. In terms of the absolute amount, the crowding-out effect on food in households without SHI was up to 5 times greater than that in households with SHI. However, the food variable was not statistically significant in households with SHI.

**Table 3:** Crowding-Out Effect of ₮10 000 OOP Health Expenditure Among Households With and Without SHI

**Consumption variables**	**Total**	**Household head have SHI**	
		**Yes**	**No**
	**Estimate (95% CI)**	**Estimate (95% CI)**	**Estimate (95% CI)**
Alcohol	-3.77 (-60.28; 52.77)	-13.49 (-67.66; 40.57)	54.55 (-213.39; 322.17)
Smoking	33.14 (-61.60; 127.7)	27.46 (-65.34; 120.47)	3.90 (-418.48; 425.96)
Clothes	-242.26 (-532.42; 47.51)	-405.77 (-705.52; -106.98)^b^	1403.26 (211.73; 2590.64)^a^
Communication	-765.30 (-871.41; -658.25)^c^	-766.24 (-879.01; -653.47)^c^	-779.68 (-1129.25; -428.45)^c^
Durable	-1493.05 (-1643.29; -1342.80)^c^	-1426.46 (-1580.67; -1272.25)^c^	-2067.53 (-2640.46; -1502.9)^c^
Education	1925.00 (1577.56; 2263.05)^c^	1869.82 (1513.20; 2226.44)^c^	2084.14 (929.97; 3246.61)^c^
Food	-432.89 (-810.37; -54.83)^a^	-301.67 (-692.02; 88.86)	-1569.33 (-3022.42; -117.9)^a^
Heating	509.89 (346.5; 673.28)^c^	479.98 (312.28; 646.72)^c^	684.19 (48.65; 1320.23)^a^
Other	-1051.70 (-1239.51; -864.84)^c^	-1050.57 (-1243.33; -852.98)^c^	-938.27 (-1552.72; -329.64)^b^
Rent	-390.63 (-599.09; -182.17)^c^	-407.69 (-628.41; -186.98)^c^	-304.73 (-1021.31; 411.01)
Transport	-1765.36 (-2028.29; -1502.44)^c^	-1725.25(-2004.76;-1455.38)^c^	-2059.23 (-2989.20;-1129.25)^c^
Utility	-175.59 (-253.53; -97.65)^c^	-168.66 (-249.63; -86.64)^c^	-200.94 (-481.59; 78.63)

Abbreviations: OOP, out-of-pocket; SHI, social health insurance. ^a^ *P* < .05; ^b^* P* < .01; ^c^*P* < .001.

Table 4 presents the crowding-out effect by income group. The crowding-out effect for food was largest for the lowest-income households (₮5149, 95% CI = −8582; −1695). The effect was more than 10 times greater than that of middle-income households, in absolute amounts, and it was statistically not significant among middle and highest-income households. Compared with their middle- and highest-income counterparts, the lowest-income households also had the largest crowding-in effect for heating. Notably, the highest-income households experienced a larger crowding-out effect for durables, and transport.

**Table 4:** Crowding-Out Effect of ₮10 000 OOP Health Expenditure Among Households by Income Level

**Consumption Variables**	**Household Income**		
	**Lowest (n = 5485)**	**Middle (n = 5485)**	**Highest (n = 5484)**
	**Estimate (95% CI)**	**Estimate (95% CI)**	**Estimate (95% CI)**
Alcohol	579.18 (34.32; 1121.50)^a^	-27.66 (-302.37; 247.39)	4.50 (-86.32; 95.34)
Smoking	747.67 (-116.36; 1616.44)	28.94 (-460.43; 518.85)	83.60 (-53.68; 220.46)
Clothes	-1084.65 (-2943.29; 773.99)	-2465.40 (-4131.91; -803.18)^b^	-241.94(-784.52; 300.63)
Communication	-1274.20 (-2048.19; -501.78)^c^	-1357.25 (-2035.88; -681.20)^c^	-546.87 (-737.28; -356.47)^c^
Durable	-1363.71 (-2164.03; -563.38)^c^	-1537.65 (-2259.23; -808.34)^c^	-1674.98 (-1975.62; -1387.23)^c^
Education	1063.58 (-605.50; 2732.68)	3006.58 (876.20; 5128.38)^b^	1574.77 (946.29; 2204.68)^c^
Food	-5149.45 (-8582.42; -1695.42)^b^	-146.03 (-2199.10; 1907.03)	178.95 (-445.23; 801.70)
Heating	2690.56 (737.14; 4649.25)^b^	564.37 (-248.25; 1374.44)	260.55 (66.42; 453.82)^b^
Other	-1700.68 (-2927.50; -472.29)^b^	75.59 (-910.56; 1056.60)	-891.89 (-1246.93; -535.42)^c^
Rent	1490.07 (-237.99; 3222.35)	152.90 (-979.28; 1279.94)	-558.32 (-916.23; -201.85)^b^
Transport	-995.14 (-2311.46; 321.18)	-2156.15 (-3650.85; -668.32)^b^	-1574.77(-2075.83; -1060.82)^c^
Utility	-162.69 (-752.93; 430.17)	-499.95 (-927.74; -70.26)^a^	-197.56 (-324.97; -68.71)^b^

Abbreviation: OOP, out-of-pocket. ^a^ *P* < .05; ^b^* P* < .01; ^c^*P* < .001.

## Discussion

 This study is the first to focus on the crowding-out effect of OOP health expenditures among Mongolian households and provides an examination of the country’s UHC progress from a different perspective. Compared with the standard strategy and impoverishment approach used previously to represent the burden of household health expenditures (such as the catastrophic expenditure approach) or the effect of healthcare expenditures on poverty levels (such as the impoverishing effects approach),^34^ assessing the impact of OOP health expenditures on household living standards by estimating the crowding-out effect allows policy-makers to approach vulnerable households in two ways. First, they can identify the part of spending that is most affected so that policies can directly target those areas, thereby increasing the marginal value per government dollar spent. Second, this information can also illuminate the overall consumption margins among households, which translates into better targeting of policy and subsidy programs for the more vulnerable. For example, supporting vulnerable households with the targeted necessities.

 If we observe crowding out in essential categories associated with the standard of living, such as food, education, transportation, or heating, this can illuminate the adverse impact of OOP health expenditures. For instance, some studies have provided evidence of a reduction (crowding-out effect) in education, and this reduction in educational expenditure may affect the household earning capacity and, in turn, the future economic status of the household.^29^ Yet, crowding-in can lead to an understanding of the most important consumption categories among households, such as transportation, which is essential for households to acquire long-distance healthcare. Rent, as another example, affects vulnerable households that do not have their own homes^29^ because housing can be considered essential for maintaining a basic standard of living.

 We discovered a significant crowding-out effect by OOP health expenditures on essential items such as food, transportation, and durable. In our sample, the lowest-income households had significant crowding-out effect on food, at the same time they spent proportionally more on food compared with the middle-income and highest-income households. Food consumption could have implications for child physiological and cognitive development in developing countries, particularly in poorer households.^30^ For the crowding out effect on clothing, we speculate that expenditures on clothing in Mongolia have a different influence on living standards compared with other countries with similar per capita incomes due to the extremely low temperatures during winter in the country. Given that households without SHI have fewer resources, crowding out for clothing is likely. This is true given that clothing does not have to be renewed every year; thus, clothing is a relatively easy expense to forgo if resources are constrained.

 We observed a crowding in effect for heating. Given that Mongolia can get extremely cold during the winter, forgoing expenditures on heating can significantly lower living standards. Heating is required in the long, cold winter season in Mongolia; the average temperature is below 0°C from November to March, and winter nights of −40°C are common most years. This may result in heating expenditures being less affected by OOP health costs.

 Social and national health insurance schemes have been introduced and developed in many LMICs with the aim of progressing toward UHC. Higher-income countries have already achieved UHC, including Australia, Canada, and New Zealand.^15^ The first countries in Asia to achieve UHC with relatively equitable access to affordable care were Japan, South Korea, and Taiwan,^16^ with Taiwan using its National Health Insurance system as the basis for a shift to true universality.^35^ However, gaps in coverage are common in LMICs and can only be met by OOP payments,^7^ general taxation, or private health insurance (PHI).^36^ Many LMICs,^1,37^ especially in Asia, face particular challenges due to remarkably limited public funds for healthcare, inefficient allocation, and overreliance on OOP payments.^38^ Mongolia is one LMIC moving toward UHC, with relatively high SHI coverage,^15^ but Mongolians also have a high share of OOP health expenditures. Mongolia faces challenges similar to those of other Asian LMICs such as Laos, Cambodia, and Myanmar, with low government investment in health, high OOP health costs, and reliance on external aid.^16^

 Our results revealed that households with SHI had almost double the OOP health expenditures of households without SHI. By crowding-out effect of OOP health expenditure among households with and without SHI, the patterns were similar between these groups. Despite the high level of enrolment, SHI may not completely succeeded on the prevention of catastrophic health expenditures and medical impoverishment.^11^ In a previous study, Mongolia had a positive Kakwani index value for OOP expenses,^39^ indicating that wealthier people or those with a higher socioeconomic status may have used more expensive services or more private sector services than do people with lower socioeconomic status.^39^

 In our sample, households in different SHI status experienced a crowding-in effect on education and heating. This contrasts with the results of other studies that identified a crowding-out effect on education.^29,30^ One of Mongolia’s most remarkable achievements of the socialist period is considered to be its progress in education,^40^ and the perceived necessity of education remains strong among Mongolian households, and hence unlikely to be crowded out by OOP health costs. Another plausible explanation for the crowding-in effect of education is that in Mongolia, only primary education is free of charge for students going to state-owned schools. Further education is OOP, and thus, crowding in can occur. Previous studies have identified inequality in college attendance between youth from low- and high-income families. The most vulnerable groups of children in Mongolia are those from urban areas and from families in the bottom-quintile income level.^40^ However, higher income households typically pay for extra informal education or tutoring. A household with high healthcare needs may thus need to rely more on this type of education, which creates crowding-in effects.

 Many households face budget constraints, often limiting their ability to afford healthcare. Large healthcare expenditures may mean the need to forgo essential items, which could be catastrophic for the survival of the members of such households.^41^ Household resource allocation decisions could adversely affect human capital investments essential for long-term potential for prosperity.^30^ Households deal with OOP health expenditure shocks through various coping strategies, such as reducing consumption expenditures, although consumption variables vary across countries and cultures.^29,42^ Health shocks thus pose a sizeable risk to households, and implementing prepayment or saving mechanisms might help protect vulnerable populations from financial threats due to illness.

 Our research had some limitations. First, our data were cross-sectional; thus, we could not determine the longitudinal trend of the effect of OOP health expenditures on spending. In developing countries, longitudinal expenditure data are often scarce.^43^ Second, our data were self-reported, opening up the possibility of recall or misreporting biases. This, however, is not unique to our study, as expenditure information often relies on self-reported surveys.^44^ Third, this dataset does not contain information on which family members (other than the household head) have SHI, and consequently, the effect(s) of SHI may be overestimated in our study. This factor, however, does not change our conclusion that SHI coverage may not prevent a crowding-out effect.

 Poorer households are more vulnerable to OOP health expenditures.^45^ According to the World Bank, 28.4% of Mongolia’s population lived below the poverty line in 2018; this translates to 905 000 Mongolians who could not afford essential goods. The results of a previous study indicated that a substantial proportion of the population faced catastrophic health expenditures and was forced into poverty as a result of making OOP payments for healthcare.^23^ Our study obtained similar results: the lowest-income households had a larger crowding-out effect for essential consumption categories such as food, communication, durables, and transportation due to OOP health expenditures. Reducing the share of OOP health expenditures, especially among low-income households, can be achieved through the provision of quality public healthcare services at a low cost for vulnerable households in Mongolia. In addition, a systematic review reported that SHI increases in service utilization could simultaneously secure financial risk protection covered populations by reducing their OOP health expenditures.^46^ The current SHI benefit is relatively low and may be insufficient to protect a person from incurring OOP health expenses in Mongolia. Increasing the benefits package of SHI is necessary, including to cover some services that are not currently covered.^23^ Another strategy that the government can consider adopting is increasing the number of private healthcare providers contracted with the SHI system, which is currently low.^47^

## Conclusion

 Empirical findings often suggest that the lowest-income households are affected more heavily by OOP health expenditures. Our study demonstrates how OOP costs can place a strain on households with limited financial resources. Our results help to clarify how households modify their consumption decisions and reallocate resources across broad commodity groups in response to OOP health expenditures. The findings suggest that OOP health expenses in Mongolia are associated with allocating household budget shares to essential expenditure items. However, allocations vary according to household income level and SHI status. We argue that the Mongolian health policy-makers must give serious consideration to reform SHI, which should be supplemented by PHI. Complementary or supplementary PHI working in tandem with SHI was suggested by some authors as a potential mechanism for eliminating the financial burden of OOP health expenses.^48^ Examining the potential of employing PHI in parallel with SHI to reduce OOP health expenditures in Mongolia remains a high priority for future research.

## Ethical issues

 This study was approved by the Institutional Review Board of National Yang-Ming University (IRB number: YM107064E-2).

## Competing interests

 Authors declare that they have no competing interests.

## Authors’ contributions

 CP and OB were responsible for conception and design. OB, TB, and DD acquired the data. CP and OB participated in analysis and interpretation of data. CP and OB drafted the manuscript. All authors critical revised the manuscript for important intellectual content.

## Funding

 This study was supported by Ministry of Science and Technology, Taiwan. Grant recipient: Christy Pu. Grant number: 109-2314-B-010 -049 -MY2.

## References

[1] Obermann K, Jowett M, Kwon S (2018). The role of national health insurance for achieving UHC in the Philippines: a mixed methods analysis. Glob Health Action.

[2] World Health Organization (WHO). The World Health Report: Health Systems Financing: The Path to Universal Coverage: Executive Summary. WHO; 2010. 10.2471/BLT.10.078741 PMC2878164 20539847

[3] Lozano R, Fullman N, Mumford JE (2020). Measuring universal health coverage based on an index of effective coverage of health services in 204 countries and territories, 1990-2019: a systematic analysis for the Global Burden of Disease Study 2019. Lancet.

[4] World Health Organization (WHO). World Health Statistics 2019: Monitoring Health for the SDGs, Sustainable Development Goals. Geneva: WHO; 2019.

[5] Wagstaff A, Eozenou P, Smitz M (2020). Out-of-pocket expenditures on health: a global stocktake. World Bank Res Obs.

[6] World Health Organization (WHO). Global Monitoring Report on Financial Protection in Health 2019. WHO; 2020.

[7] Aregbeshola BS, Khan SM (2018). Out-of-pocket payments, catastrophic health expenditure and poverty among households in Nigeria 2010. Int J Health Policy Manag.

[8] Shet N, Qadiri GJ, Kalal BS, Saldanha S (2018). Impact of out-of-pocket health care financing and health insurance utilization among the population: a systematic review. Int J Health Sci Res.

[9] Kim W, Park EC (2017). Catastrophic health expenditure status and trend of Korea in 2015. Health Policy Manag.

[10] Van Minh H, Kim Phuong NT, Saksena P, James CD, Xu K (2013). Financial burden of household out-of pocket health expenditure in Vietnam: findings from the National Living Standard Survey 2002-2010. Soc Sci Med.

[11] Li Y, Wu Q, Liu C (2014). Catastrophic health expenditure and rural household impoverishment in China: what role does the new cooperative health insurance scheme play?. PLoS One.

[12] Bayarbat U, Li Y (2020). Empirical analysis of relationship between per capita health expenditure and economic growth based on vector autoregressive model (VAR) in Mongolia. Theor Econ Lett.

[13] Zahoor Z. Socioeconomic Inequality in Primary Health Care Utilization Among the Elderly People: Evidence from Mongolia [dissertation]. University of Eastern Finland; 2019.

[14] Dugee O, Sugar B, Dorjsuren B, Mahal A (2019). Economic impacts of chronic conditions in a country with high levels of population health coverage: lessons from Mongolia. Trop Med Int Health.

[15] Erdenee O, Narula IS, Yamazaki C, Kameo S, Koyama H (2019). Mongolian Health Sector Strategic Master Plan (2006-2015): a foundation for achieving universal health coverage. Int J Health Plann Manage.

[16] Yip W. Healthcare system challenges in Asia. In: Oxford Research Encyclopedia of Economics and Finance. Oxford University Press; 2019.

[17] Onoka CA, Hanson K, Hanefeld J (2015). Towards universal coverage: a policy analysis of the development of the National Health Insurance Scheme in Nigeria. Health Policy Plan.

[18] Jigjidsuren A, Byambaa T, Altangerel E (2019). Free and universal access to primary healthcare in Mongolia: the service availability and readiness assessment. BMC Health Serv Res.

[19] Dorjdagva J, Batbaatar E, Svensson M, Dorjsuren B, Kauhanen J (2016). Catastrophic health expenditure and impoverishment in Mongolia. Int J Equity Health.

[20] Vian T, Feeley FG, Domente S, Negruta A, Matei A, Habicht J (2015). Barriers to universal health coverage in Republic of Moldova: a policy analysis of formal and informal out-of-pocket payments. BMC Health Serv Res.

[21] Taazan B, Yamamoto E, Baatar B (2020). Estimating cost of hospitalization for childbirth at a tertiary hospital in Mongolia. Nagoya J Med Sci.

[22] Bayarsaikhan D, Kwon S, Chimeddagva D (2015). Social health insurance development in Mongolia: opportunities and challenges in moving towards Universal Health Coverage. Int Soc Secur Rev.

[23] Dorjdagva J, Batbaatar E, Svensson M, Dorjsuren B, Kauhanen J (2016). Catastrophic health expenditure and impoverishment in Mongolia. Int J Equity Health.

[24] Dorj G, Sunderland B, Sanjjav T, Dorj G, Gendenragchaa B (2017). Drug pricing and reimbursement decision making systems in Mongolia. J Pharm Policy Pract.

[25] World Health Organization (WHO). Global Health Expenditure Database (GHED). WHO; 2020.

[26] Dugee O, Palam E, Dorjsuren B, Mahal A (2018). Who is bearing the financial burden of non-communicable diseases in Mongolia?. J Glob Health.

[27] Dorjdagva J. Socioeconomic-Related Health Inequalities and Health Care Utilization in Mongolia [dissertation]. University of Eastern Finland; 2017.

[28] Nguyen NM, Nguyen A (2020). Crowding-out effect of tobacco expenditure in Vietnam. Tob Control.

[29] Pal R (2013). Out-of-pocket health expenditure: impact on the consumption of Indian households. Oxf Dev Stud.

[30] Datta BK, Husain MJ, Fatehin S (2020). The crowding out effect of out-of-pocket medication expenses of two major non-communicable diseases in Pakistan. Int Health.

[31] John R, Chelwa G, Vulovic V, Chaloupka F. Using Household Expenditure Surveys for Research in the Economics of Tobacco Control. A Tobacconomics Toolkit. Chicago, IL: Tobacconomics, Health Policy Center, Institute for Health Research and Policy, University of Illinois at Chicago; 2019.

[32] Aronson JR, Johnson P, Lambert PJ (1994). Redistributive effect and unequal income tax treatment. Econ J.

[33] Koch SF (2018). Catastrophic health payments: does the equivalence scale matter?. Health Policy Plan.

[34] Arsenijevic J, Pavlova M, Groot W (2013). Measuring the catastrophic and impoverishing effect of household health care spending in Serbia. Soc Sci Med.

[35] Schmidt H, Gostin LO, Emanuel EJ (2015). Public health, universal health coverage, and Sustainable Development Goals: can they coexist?. Lancet.

[36] Odeyemi IA, Nixon J (2013). The role and uptake of private health insurance in different health care systems: are there lessons for developing countries?. Clinicoecon Outcomes Res.

[37] Erlangga D, Suhrcke M, Ali S, Bloor K (2019). The impact of public health insurance on health care utilisation, financial protection and health status in low- and middle-income countries: a systematic review. PLoS One.

[38] Dieleman J, Campbell M, Chapin A (2017). Evolution and patterns of global health financing 1995-2014: development assistance for health, and government, prepaid private, and out-of-pocket health spending in 184 countries. Lancet.

[39] Bayati M, Mehrolhassani MH, Yazdi-Feyzabadi V (2019). A paradoxical situation in regressivity or progressivity of out of pocket payment for health care: which one is a matter of the health policy maker’s decision to intervention?. Cost Eff Resour Alloc.

[40] Banzragch O, Mizunoya S, Bayarjargal M (2019). Education inequality in Mongolia: measurement and causes. Int J Educ Dev.

[41] Koch SF, Setshegetso N (2020). Catastrophic health expenditures arising from out-of-pocket payments: evidence from South African income and expenditure surveys. PLoS One.

[42] Somi MF, Butler JR, Vahid F, Njau JD, Abdulla S (2009). Household responses to health risks and shocks: a study from rural Tanzania raises some methodological issues. J Int Dev.

[43] United Nations Statistics Division. Household Sample Surveys in Developing and Transition Countries. Vol 96. New York: United Nations Publications; 2005.

[44] Bhandari A, Wagner T (2006). Self-reported utilization of health care services: improving measurement and accuracy. Med Care Res Rev.

[45] Ssewanyana S, Kasirye I (2020). Estimating Catastrophic Health Expenditures from Household Surveys: Evidence from Living Standard Measurement Surveys (LSMS)-Integrated Surveys on Agriculture (ISA) from Sub-Saharan Africa. Appl Health Econ Health Policy.

[46] Khan JA, Ahmed S, Sultana M (2020). The effect of a community-based health insurance on the out-of-pocket payments for utilizing medically trained providers in Bangladesh. Int Health.

[47] Munkh-Erdene L. An Assessment of Mongolian Health Insurance Coverage Among Two Vulnerable Groups: Herdsmen and Students [dissertation]. American University of Armenia (AUA); 2014.

[48] Grigorakis N, Floros C, Tsangari H, Tsoukatos E (2016). Out of pocket payments and social health insurance for private hospital care: Evidence from Greece. Health Policy.

